# Length-dependent changes in contractile dynamics are blunted due to cardiac myosin binding protein-C ablation

**DOI:** 10.3389/fphys.2014.00461

**Published:** 2014-12-02

**Authors:** Ranganath Mamidi, Kenneth S. Gresham, Julian E. Stelzer

**Affiliations:** Department of Physiology and Biophysics, School of Medicine, Case Western Reserve UniversityCleveland, OH, USA

**Keywords:** cMyBP-C, length-dependent activation, sarcomere length, myofilament function, cross-bridge kinetics

## Abstract

Enhanced cardiac contractile function with increased sarcomere length (SL) is, in part, mediated by a decrease in the radial distance between myosin heads and actin. The radial disposition of myosin heads relative to actin is modulated by cardiac myosin binding protein-C (cMyBP-C), suggesting that cMyBP-C contributes to the length-dependent activation (LDA) in the myocardium. However, the precise roles of cMyBP-C in modulating cardiac LDA are unclear. To determine the impact of cMyBP-C on LDA, we measured isometric force, myofilament Ca^2+^-sensitivity (pCa_50_) and length-dependent changes in kinetic parameters of cross-bridge (XB) relaxation (*k*_rel_), and recruitment (*k*_df_) due to rapid stretch, as well as the rate of force redevelopment (*k*_tr_) in response to a large slack-restretch maneuver in skinned ventricular multicellular preparations isolated from the hearts of wild-type (WT) and cMyBP-C knockout (KO) mice, at SL's 1.9 μm or 2.1 μm. Our results show that maximal force was not significantly different between KO and WT preparations but length-dependent increase in pCa_50_ was attenuated in the KO preparations. pCa_50_ was not significantly different between WT and KO preparations at long SL (5.82 ± 0.02 in WT vs. 5.87 ± 0.02 in KO), whereas pCa_50_ was significantly different between WT and KO preparations at short SL (5.71 ± 0.02 in WT vs. 5.80 ± 0.01 in KO; *p* < 0.05). The *k*_tr_, measured at half-maximal Ca^2+^-activation, was significantly accelerated at short SL in WT preparations (8.74 ± 0.56 s^−1^ at 1.9 μm vs. 5.71 ± 0.40 s^−1^ at 2.1 μm, *p* < 0.05). Furthermore, *k*_rel_ and *k*_df_ were accelerated by 32% and 50%, respectively at short SL in WT preparations. In contrast, *k*_tr_ was not altered by changes in SL in KO preparations (8.03 ± 0.54 s^−1^ at 1.9 μm vs. 8.90 ± 0.37 s^−1^ at 2.1 μm). Similarly, KO preparations did not exhibit length-dependent changes in *k*_rel_ and *k*_df_. Collectively, our data implicate cMyBP-C as an important regulator of LDA via its impact on dynamic XB behavior due to changes in SL.

## Introduction

Length-dependent activation (LDA) is the mechanism by which force production in the heart becomes more sensitive to Ca^2+^ as the sarcomere length (SL) is increased (Allen and Kentish, 1985). Although it is well recognized that LDA underlies the Frank-Starling's Law of the heart, the cellular and molecular mechanisms that modulate this process are still poorly understood mainly because LDA involves a dynamic and complex interplay between a multitude of thick- and thin-filament-based mechanisms (De Tombe et al., 2010). The thick-filament-based mechanisms involve augmentation of strong crossbridge (XB) formation followed by enhancement in the myofilament Ca^2+^ sensitivity upon a reduction in the myofilament lattice spacing and the radial distance between the thick and thin filaments at long SL (Fuchs and Smith, 2001). The strongly-bound XBs then cooperatively recruit additional near-neighbor XBs into the force-bearing state (Gordon et al., 2000; Regnier et al., 2004). The thin-filament-based mechanisms involve an increased affinity of troponin C (TnC) to Ca^2+^ when the neighboring TnC sites are bound with Ca^2+^ and the increased affinity of TnC to Ca^2+^ is also a result of a positive feedback effect of the strongly-bound XBs (Hannon et al., 1992; Moss et al., 2004; Li et al., 2014). Furthermore, the cooperative effect between neighboring troponin-tropomyosin (Tn-Tm) complexes also impacts the Ca^2+^ binding properties of the thin-filament (Butters et al., 1997; Farman et al., 2010) and thus influence the LDA in cardiac muscle (for details on LDA refer to reviews by Konhilas et al., 2002; Hanft et al., 2008; De Tombe et al., 2010; Campbell, 2011).

Earlier investigations have proposed that LDA in cardiac muscle is influenced by various sarcomeric proteins such as TnC (Gulati et al., 1991), TnI (Konhilas et al., 2003; Tachampa et al., 2007), TnT (Chandra et al., 2006), myosin heavy chain (Korte and McDonald, 2007), essential light chain (Michael et al., 2013), and titin (Fukuda et al., 2003). In addition to the aforementioned sarcomeric proteins, it is also possible that cardiac myosin binding protein-C (cMyBP-C) may be an important modulator of cardiac LDA because cMyBP-C is uniquely positioned in the sarcomere to interact with both the thick- and thin-filaments (Squire et al., 2003; Shaffer et al., 2009; Previs et al., 2012; Sadayappan and De Tombe, 2012; Mun et al., 2014), and has been shown to be important in regulating key aspects of dynamic XB behavior (Stelzer et al., 2006a,b; Coulton and Stelzer, 2012), and providing structural rigidity to the myofilament lattice (Palmer et al., 2011).

Importantly, recent evidence from low-angle X-ray diffraction experiments showed that cMyBP-C tethers the myosin XBs closer to the thick-filament backbone and that ablation of cMyBP-C results in the radial displacement of XBs closer to the thin-filament (Colson et al., 2007). The role of cMyBP-C in LDA is also underscored by the observation that length-dependent increase in myofilament Ca^2+^ sensitivity was blunted in cardiac preparations from patients with cMyBP-C mutations (Van Dijk et al., 2012; Sequeira et al., 2013). However, the precise roles of cMyBP-C in modulating length-dependent changes in cardiac contractile dynamics are still unknown. Therefore, to determine the impact of cMyBP-C on length-dependent changes in contractile dynamics, we utilized skinned myocardium from a cMyBP-C knock-out (KO) mouse model (Harris et al., 2002), and measured steady-state contractile parameters and we also used stretch activation experiments to measure the kinetic parameters. We measured Ca^2+^-activated maximal force, myofilament Ca^2+^ sensitivity (pCa_50_), rate of force redevelopment (*k*_tr_), rate of XB relaxation (*k*_rel_), and rate of XB recruitment (*k*_df_) at short (1.9 μm) and at long (2.1 μm) SL's. Our results show that the length-dependent increase in pCa_50_ was attenuated in the KO preparations compared to wild-type (WT) preparations. Furthermore, length-dependent changes in dynamic contractile parameters *k*_tr_, *k*_rel_, and *k*_df_ were blunted in KO preparations compared to WT preparations, indicating that cMyBP-C plays a critical role in the myofilament-mediated response in cardiac LDA.

## Materials and methods

### Ethical approval and animal treatment protocols

This study was performed according to the protocols laid out in the *Guide for the Care and Use of Laboratory Animals* (NIH Publication No. 85–23, Revised 1996), and was conducted according to the guidelines of the Institutional Animal Care and Use Committee at Case Western Reserve University. Mice aged 3–6 months, of both sexes, and belonging to SV/129 strain were used for the experiments. KO mice used in this study were previously generated and well-characterized (Harris et al., 2002). WT mice expressing normal, full-length cMyBP-C in the myocardium were used as controls.

### Estimation of cMyBP-C content and phosphorylation status of sarcomeric proteins in WT and KO heart samples

Cardiac myofibrils were isolated from frozen mouse ventricles on the day of the experiment (Gresham et al., 2014). A piece of the frozen tissue was thawed in a fresh relaxing solution, homogenized, and the myofibrils were then skinned for 15 min with 1% Triton X-100 (Cheng et al., 2013). Skinned myofibrils were then resuspended in fresh relaxing solution containing protease and phosphatase inhibitors (PhosSTOP and cOmplete ULTRA Tablets; Roche Applied Science, Indianapolis, IN, USA) and stored on ice. To determine the cMyBP-C content and myofilament protein phosphorylation status, ventricular samples were solubilized by adding Laemmli buffer and were heated to 90°C for 5 min. For Western blot analysis, 10 μg of cardiac myofibrils were electrophoretically separated on 4–20% Tris-glycine gels (Lonza Walkersville Inc., Rockland, ME, USA) at 180 V for 60 min. Proteins were transferred to PVDF membranes and incubated overnight with a primary antibody that detects cMyBP-C (Santa Cruz Biotechnology, Santa Cruz, CA, USA) as described previously (Cheng et al., 2013). For Pro-Q phosphoprotein analysis, 2.5 μg of solubilized cardiac myofibrils were electrophoretically separated at 180 V for 85 min then fixed and stained with Pro-Q diamond phosphoprotein stain (Invitrogen, Carlsbad, CA, USA) to assess the phosphorylation status of sarcomeric proteins. After imaging the Pro-Q stained gels, the gels were counterstained with Coomassie blue to determine if there are any changes in the isoform expression of sarcomeric proteins. Densitometric scanning of the stained gels was done using Image J software (U.S. National Institutes of Health, Bethesda, MD, USA) (Gresham et al., 2014).

### Preparation of skinned ventricular multicellular preparations and Ca^2+^ solutions for experiments

Skinned ventricular multicellular preparations were prepared as described previously (Cheng et al., 2013; Gresham et al., 2014). In brief, ventricular tissue was homogenized in a relaxing solution and skinned for 60 min using 1% Triton-X 100. Multicellular preparations with dimensions ~100 μm in width and 400 μm in length were selected for the experiments. The composition of various Ca^2+^ activation solutions used for the experiments was calculated using a computer program (Fabiato, 1988) and known stability constants (Godt and Lindley, 1982). All solutions contained the following (in mM): 100 N, N-bis (2-hydroxyethyl)-2-aminoethanesulfonic acid (BES), 15 creatine phosphate, 5 dithiothreitol, 1 free Mg^2+^, and 4 MgATP. The maximal activating solution (pCa 4.5; pCa = -log [Ca^2+^]_free_) also contained 7 EGTA and 7.01 CaCl_2_; while the relaxing solution (pCa 9.0) contained 7 EGTA and 0.02 CaCl_2_; and the pre-activating solution contained 0.07 EGTA. The pH of the Ca^2+^ solutions was set to 7.0 with KOH and the ionic strength was 180 mM. A range of pCa solutions, containing varying amounts of [Ca^2+^]_free_, were then prepared by mixing appropriate volumes of pCa 9.0 and 4.5 stock solutions and the experiments were performed at 22°C.

### Experimental apparatus for the estimation of isometric force and force-pCa relationships

Detergent-skinned ventricular preparations were held between a motor arm (312C; Aurora Scientific Inc., Aurora, Ontario, Canada) and a force transducer (403A; Aurora Scientific Inc.) as described previously (Merkulov et al., 2012; Cheng et al., 2013). Changes in the motor position and signals from the force transducer were sampled (16-bit resolution, DAP5216a, Microstar Laboratories; Bellevue, WA) at 2.0 kHz using SL control software (Campbell and Moss, 2003). As previously described (Stelzer et al., 2006a,b,c), the experimental set up was positioned on the stage of an inverted microscope (Olympus; Tokyo, Japan) that was fitted with a 40X objective and a closed-circuit television camera (model WV-BL600; Panasonic, Tokyo, Japan). To illuminate the multicellular preparations, we used the light emanating from a halogen lamp and the light was passed through a cut-off filter (transmission >620 nm) before reaching the preparation. Bitmap images of the preparations were captured using an AGP 4X/2X graphics card and its associated software (ATI Technologies) to assess the SL of our preparations during the experiment. For all mechanical measurements, SL of the muscle preparations was set to 1.9 or 2.1 μm in a relaxing solution and submaximal force (P) developed at each pCa was normalized to maximal force (P_o_, at pCa 4.5) i.e., P/P_o_ to construct the force-pCa relationships (Desjardins et al., 2012; Cheng et al., 2013). SL of the preparations was initially set using a high definition video camera and large video monitor, and was also assessed at the end of experiments to make sure that SL did not deviate from the initial SL following Ca^2+^-activation. We chose this specific range of SL for our experiments because this range falls within the well-characterized working range (~1.8–2.3 μm) of the sarcomeres in the heart muscle (Pollack and Huntsman, 1974; Rodriguez et al., 1992; Granzier and Irving, 1995; Hanft et al., 2008). The apparent cooperativity of force development was estimated from the steepness of a Hill plot transformation of the force-pCa relationships. The force-pCa data were fit using the equation P/P_o_ = [Ca^2+^]*^nH^*/(*k^nH^* + [Ca^2+^]*^nH^*), where *n_H_* is the Hill coefficient and *k* is the pCa required to produce half-maximal activation (i.e., pCa_50_) (Gresham et al., 2014).

### Measurement of the rate of force redevelopment (*k_tr_*)

*k*_tr_ was measured at 50% of maximal activation in WT and KO muscle preparations to assess the rate of XB transitions from weak- to strong-binding states (Brenner and Eisenberg, 1986; Campbell, 1997). Measurement of *k*_tr_ in Ca^2+^-activated muscle preparations was performed according to a mechanical slack-restretch protocol described previously (Stelzer et al., 2006b; Chen et al., 2010; Cheng et al., 2013). Skinned muscle preparations were transferred from relaxing (pCa 9.0) to an activating Ca^2+^ solution (pCa ranging from 6.2 to 4.5), and once the muscle preparations attained steady-state isometric force, they were rapidly slackened by 20% of their original muscle length and were held for 10 ms using a high-speed length control device (Aurora Scientific Inc.). The slackening was followed by the brief period of unloaded shortening which resulted in a rapid decline in force due to detachment of the strongly-bound XBs. The muscle preparation was then rapidly restretched back to its original length and the time course of force redevelopment was measured. *k*_tr_ for each slack-restretch maneuver was estimated by linear transformation of the half-time of force redevelopment, i.e., *k*_tr_ = 0.693/*t*_1/2_, as described previously (Chen et al., 2010; Cheng et al., 2013; Gresham et al., 2014).

### Stretch activation experiments to measure dynamic contractile parameters

**Figure 1:**
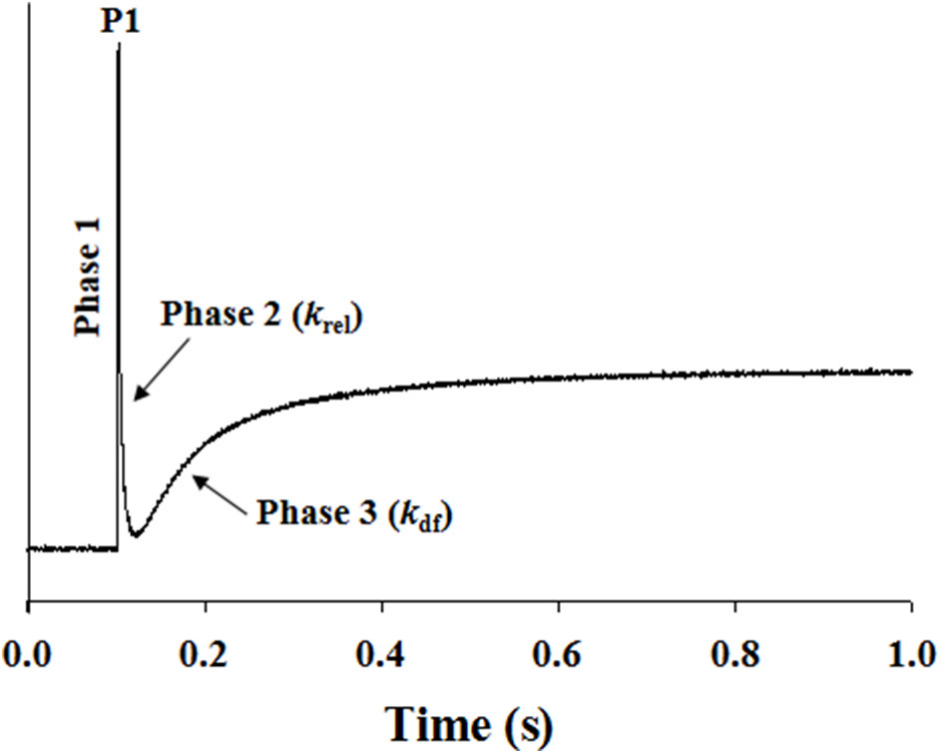
**Representative stretch activation response in a WT cardiac muscle preparation**. Shown is a typical force response evoked by a sudden 2% stretch in muscle length (ML) in an isometrically-contracting WT muscle preparation set to a SL of 2.1 μm. Highlighted are the important phases of the force response and various stretch activation parameters that are derived from the response. Phase 1 represents the immediate increase in force in response to the sudden stretch in ML. P1 is the magnitude of the immediate force response and is measured from the pre-stretch isometric steady-state force to the peak of phase 1. Phase 2 represents the rapid decline in the force with a dynamic rate constant *k*_rel_, an index of the XB detachment rate. Phase 3 represents the delayed force development with a dynamic rate constant *k*_df_, an index of the XB recruitment rate (please see methods for additional details).

Stretch activation experiments were carried out as previously described (Cheng et al., 2013; Gresham et al., 2014), except that in this study a 2% stretch of initial muscle length perturbation was utilized. Muscle preparations were placed in pCa solutions that produced submaximal force (~50% of maximal force), and were allowed to develop a steady-state force. Muscle preparations were then rapidly stretched by 2% of their initial muscle length and were held at the increased length for 5 s before being returned back to their initial muscle length. The characteristic features of the stretch activation response in cardiac muscle have been described previously (Stelzer et al., 2006d; Ford et al., 2010), and the stretch activation parameters measured are presented in Figure 1. In brief, a sudden 2% stretch of muscle length elicits an instantaneous rise in force (P1) in the muscle preparation, which is due to the strain of elastic elements within the strongly-bound XBs (Phase 1). The force then rapidly declines because the strained XBs rapidly detach (Phase 2) and equilibrate into a non-force producing state, with a characteristic rate constant *k*_rel_. Following this phase of rapid decline, force development occurs gradually (Phase 3), with a characteristic rate constant *k*_df_, which is a result of length-induced recruitment of new XBs into the force-bearing state (Stelzer et al., 2006d; Gresham et al., 2014). Stretch activation amplitudes were normalized to prestretch Ca^2+^-activated force and were measured as described previously (Desjardins et al., 2012; Gresham et al., 2014). *k*_rel_ and *k*_df_ were estimated using a linear transformation of the half time of force decay and force redevelopment.

### Data analysis

Data were analyzed using Two-Way analysis of variance (ANOVA). One factor in this analysis was cMyBP-C variant (WT or KO), and the second was SL (1.9 or 2.1 μm). Therefore, we used Two-Way ANOVA to test the hypothesis that the effect of the SL on a given contractile parameter depended on the cMyBP-C variant (interaction effect). When the interaction effect was significant, it showed that the effects of SL on various contractile parameters were different in the presence or absence of cMyBP-C. When the interaction effect was not significant, we interpreted the main effect due to cMyBP-C variant or SL. Planned multiple pairwise comparisons were made using Fisher's LSD method (Mamidi and Chandra, 2013; Mamidi et al., 2013a,b) to test the effects of cMyBP-C variant or SL on various contractile parameters. Values are reported as mean ± s.e.m. The criterion for statistical significance was set at *P* < 0.05. Asterisks in figures and tables represent statistical significance using *post-hoc* (Fisher's LSD) comparisons.

## Results

### Effect of ablation of cMyBP-C on the expression and phosphorylation levels of sarcomeric proteins

**Figure 2:**
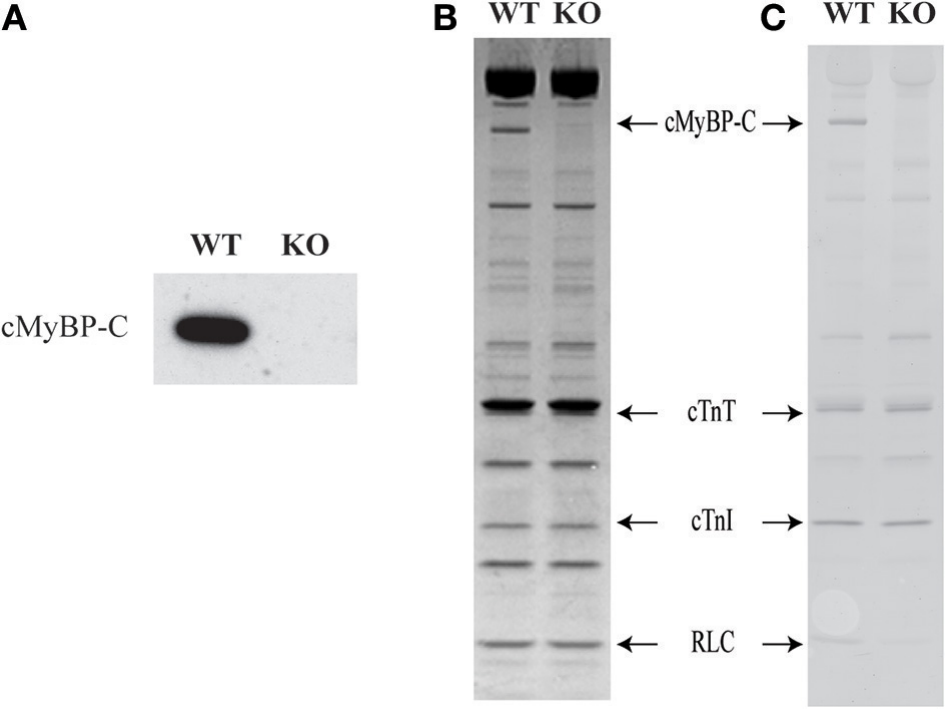
**Western blot, SDS-PAGE and Pro-Q analysis to assess the sarcomeric protein expression and phosphorylation levels in WT and KO hearts**. Ventricular myofibrils from WT and KO hearts were run on a 4–20% tris-glycine gel. **(A)** Western blot showing the presence of cMyBP-C in WT but not in the KO heart sample. **(B)** Coomassie blue-stained SDS gel showing the complete absence of cMyBP-C in the KO hearts. **(C)** Pro-Q Diamond-stained SDS gel showing that there are no changes in the phosphorylation status of sarcomeric proteins between WT and KO samples. As expected, a Pro-Q band corresponding to cMyBP-C was absent in the lane containing KO sample. WT, wild-type; KO, knock-out; cMyBP-C, cardiac myosin binding protein-C; cTnT, cardiac troponin T; cTnI, cardiac troponin I; RLC, regulatory light chain.

Western blot analysis of WT and KO ventricular samples was done using a primary antibody that detects cMyBP-C protein (Cheng et al., 2013). As predicted, cMyBP-C is present in the WT sample but is completely absent in the KO sample (Figure 2A). SDS gels loaded with ventricular samples from WT and KO hearts were stained with Coomassie blue or Pro-Q Diamond stain to assess the effects of cMyBP-C KO on sarcomeric protein isoform expression and phosphorylation levels (Figures 2B,C, respectively). As reported in our recent studies (Desjardins et al., 2012; Merkulov et al., 2012) the KO hearts exhibited a slight increase (16 ± 3%) in the level of β-myosin heavy chain (MHC) expression (data not shown). Consistent with our previous studies (Desjardins et al., 2012; Merkulov et al., 2012), the expression and phosphorylation levels of other regulatory contractile sarcomeric proteins such as cardiac TnT, cardiac TnI, and regulatory light chain were not different between WT and KO skinned myocardium (Figures 2B,C).

### Effect of cMyBP-C on length-dependent changes in Ca^2+^-activated maximal force production

To assess the effect of cMyBP-C on length-dependent changes in thin-filament activation, Ca^2+^-activated maximal force production (at pCa 4.5) was measured at SL's 1.9 and 2.1 μm in WT and KO muscle preparations (values are shown in Table 1). Two-Way ANOVA (see Data analysis under Methods section for details) revealed no significant interaction effect, but revealed a significant main effect (*P* < 0.005) of SL on Ca^2+^-activated maximal force production. To probe the determining factor for the significant main effect, subsequent *post-hoc* tests were carried out. These *post-hoc* tests using multiple planned pairwise comparisons showed that maximal force production was not significantly different between WT and KO groups at either SL (Table 1). However, maximal force (F_max_) was significantly decreased by ~34% and ~38% at short SL vs. long SL in WT and KO groups, respectively (Table 1). Similar trends were observed regarding the Ca^2+^-independent forces measured at pCa 9.0 (F_min_) in WT and KO groups (Table 1). Collectively, our results demonstrate that cMyBP-C does not impact the length-dependent changes in Ca^2+^-activated maximal force and Ca^2+^-independent force production.

**Table 1:** **Steady-state mechanical properties of WT and KO ventricular multicellular preparations**.

**Group**	**F_min_ (mN/mm^2^)**	**F_max_ (mN/mm^2^)**	***n*_H_**	**pCa_50_**
**SL 1.9 μm**				
WT	0.82 ± 0.12^*^	17.29 ± 1.98^*^	3.41 ± 0.32^*^	5.71 ± 0.02^*^
KO	0.90 ± 0.13^*^	14.93 ± 1.56^*^	2.30 ± 0.08^†^	5.80 ± 0.01^*^^†^
**SL 2.1 μm**				
WT	2.14 ± 0.28	26.32 ± 2.93	2.47 ± 0.22	5.82 ± 0.02
KO	1.97 ± 0.25	23.98 ± 2.58	2.49 ± 0.23	5.87 ± 0.02

*F_min_: Ca^2+^-independent force measured at pCa 9.0; F_max_: maximal Ca^2+^-activated force measured at pCa 4.5; n_H_: Hill coefficient of the force-pCa relationship; pCa_50_: pCa value required for the generation of half-maximal force. Values are expressed as mean ± s.e.m., from 7 to 17 multicellular preparations and 3 to 5 hearts per each group*.

* *Significantly different from the corresponding group at SL 2.1 μm; P < 0.05*.

† *Significantly different from the corresponding WT group at SL 1.9 μm, P < 0.05*.

### Effect of cMyBP-C on length-dependent changes in myofilament Ca^2+^ sensitivity (pCa_50_) and cooperativity of force development (*n*_H_)

**Figure 3:**
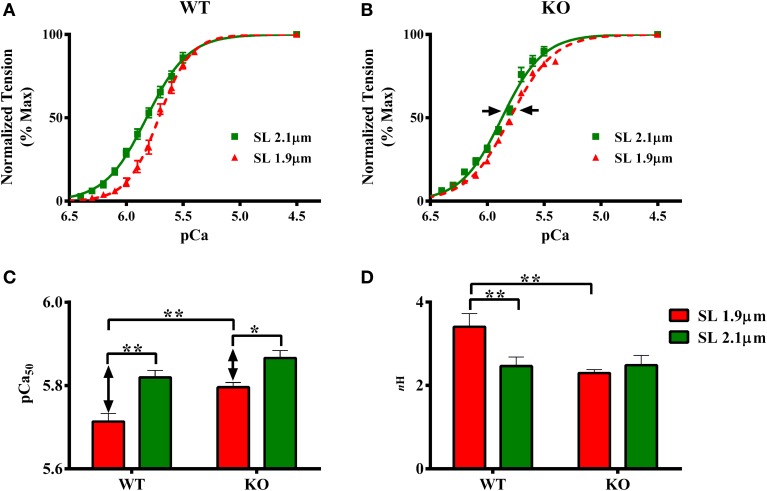
**Effect of cMyBP-C on length-dependent changes in myofilament Ca^2+^ sensitivity (pCa_50_) and cooperativity of force production (*n*_H_)**. Force-pCa relationships were constructed by plotting normalized forces generated at a range of pCa. The Hill equation was then fitted to force-pCa relationships to estimate pCa_50_ and *n*_H_ values in WT and KO groups at SL's 1.9 and 2.1 μm. **(A)** Effect of cMyBP-C on the force-pCa relationships in WT group at SL's 1.9 and 2.1 μm. **(B)** Effect of cMyBP-C on the force-pCa relationships in KO group at SL's 1.9 and 2.1 μm. **(C)** Effect of cMyBP-C on pCa_50_ in WT and KO groups at SL's 1.9 and 2.1 μm. **(D)** Effect of cMyBP-C on *n*_H_ in WT and KO groups at SL's 1.9 and 2.1 μm. Two-Way ANOVA revealed no significant interaction effect, but revealed significant main effects of SL (*P* < 0.005) and cMyBP-C (*P* < 0.005) on pCa_50_. Subsequent *post-hoc* tests revealed that pCa_50_ was significantly higher at 2.1 μm as indicated by a leftward shift in the force-pCa relationships in both WT and KO groups. Also, the SL-dependent increase in pCa_50_(ΔpCa_50_) was attenuated in KO group (indicated by arrows in **B, C**). Two-Way ANOVA revealed a significant interaction effect (*P* < 0.05) on *n*_H_ suggesting that cMyBP-C influences the effect of SL on *n*_H_. Subsequent *post-hoc* tests revealed that *n*_H_ significantly increased at SL 1.9 μm in WT but not in KO group. Determinations were made from 7 to 10 multicellular preparations and 3 to 4 hearts per each group. Values are reported as mean ± s.e.m. ^*^P < 0.05; ^**^P < 0.005.

The effect of cMyBP-C on length-dependent changes in pCa_50_ was assessed by plotting normalized force values against a range of pCa and constructing force-pCa relationships at SL's 1.9 and 2.1 μm in WT and KO groups. pCa_50_, the pCa required to generate half-maximal force, was estimated by fitting the Hill equation to the force-pCa relationships (Figures 3A,B, Table 1). Two-Way ANOVA revealed no significant interaction effect, but revealed significant main effects of SL (*P* < 0.005) and cMyBP-C (*P* < 0.005) on pCa_50_. Subsequent *post-hoc* tests revealed that the main effect of SL was because of the following: pCa_50_ significantly increased upon increasing the SL from 1.9 to 2.1 μm as indicated by a leftward shift in the force-pCa relationships in both WT and KO groups (Figures 3A,B). The SL-dependent increase in pCa_50_ (ΔpCa_50_) was attenuated in KO group when compared to ΔpCa_50_ of WT group (Figure 3B). In WT group ΔpCa_50_ was 0.11 pCa units whereas in the KO group ΔpCa_50_ was 0.07 pCa units. This attenuation of pCa_50_ can be attributed to the fact that KO group exhibited a significantly higher pCa_50_ at SL 1.9 μm compared to WT group (Figure 3C; Table 1), indicating that cardiac thin-filaments are more sensitive to Ca^2+^ activation at short SL in the KO group. Collectively, our results demonstrate that cMyBP-C impacts mechanisms that underlie length-dependent increases in myofilament Ca^2+^ sensitivity.

The effect of cMyBP-C on length-dependent changes in *n*_H_ was assessed by fitting Hill's equation to the force-pCa relationships constructed at SL's 1.9 and 2.1 μm in WT and KO groups (Figure 3D; Table 1). Two-Way ANOVA revealed a significant interaction effect (*P* < 0.05) on *n*_H_ suggesting that cMyBP-C influenced the effect of SL on *n*_H_. Subsequent *post-hoc* tests revealed that *n*_H_ significantly increased by ~38% at SL 1.9 μm in WT group, a result that agrees with earlier studies (Ford et al., 2012; Gollapudi et al., 2012). However, such an increase in *n*_H_ at SL 1.9 μm was not observed in KO group (Figure 3D)—suggesting that the absence of cMyBP-C impairs length-dependent changes in cooperative mechanisms in the sarcomere.

### Effect of cMyBP-C on length-dependent changes in the rate of force redevelopment (*k_tr_*)

**Figure 4:**
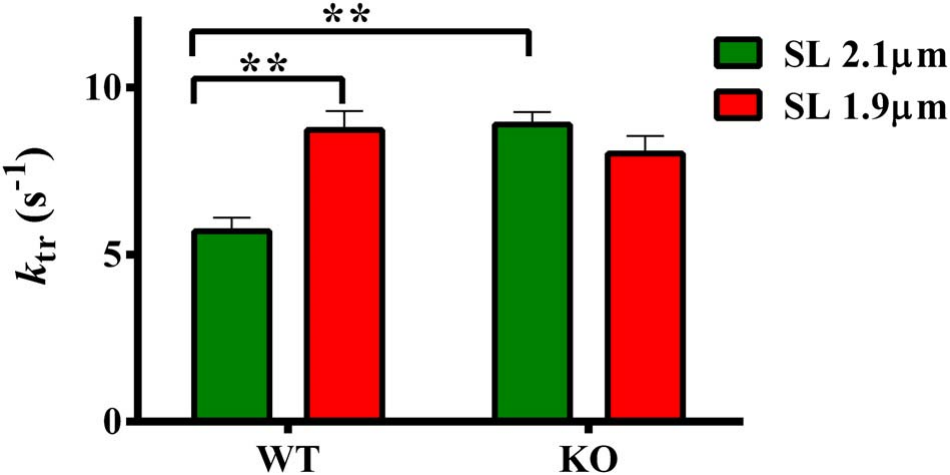
**Effect of cMyBP-C on length-dependent changes in the rate of force redevelopment (*k*_tr_)**. *k*_tr_ was measured at 50% level of activation in WT and KO groups at SL's 2.1 and 1.9 μm using a mechanical slack-restretch protocol (Gresham et al., 2014). Two-Way ANOVA revealed a significant interaction effect (*P* < 0.005) on *k*_tr_ and *post-hoc* tests showed that *k*_tr_ significantly accelerated by ~53% at short SL vs. long SL in WT group. However, such a trend was absent in the KO group. Furthermore, *k*_tr_ significantly accelerated by ~56% in KO vs. WT group at long SL. Determinations were made from 6 to 13 multicellular preparations and 3 to 4 hearts per each group. Values are reported as mean ± s.e.m. ^**^P < 0.005.

*k*_tr_ is a measure of XB transition rate from a weakly- to a strongly-bound XB state (Brenner and Eisenberg, 1986; Campbell, 1997). We have previously shown that ablation of cMyBP-C accelerates submaximal *k*_tr_ at long SL (Stelzer et al., 2006b)—indicating that KO group exhibited an accelerated rate of XB turnover from weak- to strong-bindings states. We now sought to determine whether such effects are also observed at short SL in the KO group. Therefore, we measured *k*_tr_ at 1.9 and 2.1 μm to gain insights into the effect of cMyBP-C on length-dependent changes in *k*_tr_. Two-Way ANOVA revealed a significant interaction effect (*P* < 0.005) on *k*_tr_ suggesting that cMyBP-C influenced the effect of SL on *k*_tr_. The cause of the interaction effects was assessed by *post-hoc* multiple pairwise comparisons which showed that submaximal *k*_tr_ was accelerated in KO compared to WT group at long SL (Figure 4; Table 2) as reported earlier (Stelzer et al., 2006b). Furthermore, *k*_tr_ was significantly accelerated by ~53% at short SL compared to long SL in WT group (Figure 4; Table 2). However, an acceleration of *k*_tr_ at short SL was absent in KO group (Figure 4) such that differences in *k*_tr_ between WT and KO groups observed at long SL were no longer apparent at short SL. These results indicate that cMyBP-C mediates the length-dependent changes in XB turnover rate.

**Table 2:** **Dynamic contractile parameters of WT and KO ventricular multicellular preparations**.

**Group**	***k*_tr_ (s^−1^)**	***k*_rel_ (s^−1^)**	***k*_df_ (s^−1^)**	**P1**
**SL 1.9 μm**				
WT	8.74 ± 0.56^*^	45.01 ± 4.06^*^	12.26 ± 1.53^*^	0.535 ± 0.022^*^
KO	8.03 ± 0.54	36.96 ± 2.94	9.64 ± 0.39	0.543 ± 0.048
**SL 2.1 μm**				
WT	5.71 ± 0.40	34.21 ± 2.12	8.17 ± 0.52	0.604 ± 0.016
KO	8.90 ± 0.37^†^	45.76 ± 4.01^†^	11.27 ± 0.67^†^	0.528 ± 0.026^†^

*k_tr_: rate force redevelopment; k_rel_: rate of force decay; k_df_: rate of force development; P_1_: the magnitude of peak force attained following a rapid stretch of muscle length in an isometrically-activated muscle preparation. k_rel_ and k_df_ were estimated using a linear transformation of the half time of force decay and force redevelopment. Values are expressed as mean ± s.e.m., from 6 to 13 multicellular preparations and 3 to 4 hearts per each group*.

* *Significantly different from the corresponding WT group at SL 2.1 μm; P < 0.05*.

† *Significantly different from the corresponding WT group at SL 2.1 μm, P < 0.05*.

### Effects of cMyBP-C on length-dependent changes in the rates of stretch-induced XB relaxation (*k_rel_*) and XB recruitment (*k_df_*)

Our data shows that cMyBP-C affects the length-dependent changes in the XB turnoverrate, *k*_tr_ (Figure 4). Because *k*_tr_ is proportional to the sum of *f* (rate of XB attachment) + *g* (rate of XB detachment) according to a two-state XB model (Brenner, 1988), we sought to determine if the effect of cMyBP-C on length-dependent changes in *k*_tr_ were due to changes in either the rate of XB detachment or the rate of XB attachment kinetics, or both. We used stretch activation experiments (described in the methods section) to measure *k*_rel_ and *k*_df_ which are measures of the rates of XB detachment and XB recruitment, respectively (Cheng et al., 2013; Gresham et al., 2014).

**Figure 5:**
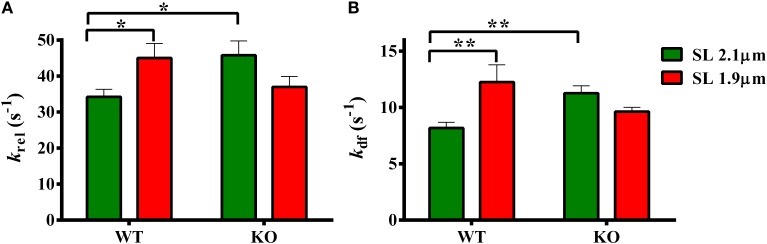
**Effect of cMyBP-C on length-dependent changes in the rates of XB detachment (*k*_rel_) and XB recruitment (*k*_df_)**. Isometrically-activated ventricular preparations were subjected to a sudden 2% stretch in their muscle length and the elicited force responses were used to estimate **(A)** *k*_rel_ and **(B)** *k*_df_ in WT and KO groups at SL's 2.1 and 1.9 μm as described in the methods section (Cheng et al., 2013; Gresham et al., 2014). Two-Way ANOVA revealed a significant interaction effect (*P* < 0.05) on *k*_rel_ and *post-hoc* tests showed that *k*_rel_ significantly accelerated by ~32% at short SL vs. long SL in WT group but such a trend was absent in KO group. *k*_rel_ significantly accelerated by ~34% in KO vs. WT group at long SL **(A)**. Two-Way ANOVA revealed a significant interaction effect (*P* < 0.005) on *k*_df_ and *post-hoc* tests showed that *k*_df_ significantly accelerated by ~50% at short SL vs. long SL in WT group but such a trend was absent in KO group. In addition, *k*_df_ significantly accelerated by ~39% in KO vs. WT group at long SL **(B)**. Determinations were made from 6 to 13 multicellular preparations and 3 to 4 hearts per each group. Values are reported as mean ± s.e.m. ^*^P < 0.05; ^**^P < 0.005.

Two-Way ANOVA revealed a significant interaction effect (*P* < 0.05) on *k*_rel_ suggesting that cMyBP-C influenced the effect of SL on the rate of XB detachment kinetics. The cause of the interaction effect was evident from the *post-hoc* multiple pairwise comparisons which revealed that *k*_rel_ was significantly accelerated by ~32% at short SL in WT group but such an acceleration of *k*_rel_ at short SL was absent in KO group (Figure 5A; Table 2). Furthermore, in agreement with recent studies (Stelzer et al., 2006a; Merkulov et al., 2012), our data shows that *k*_rel_ was significantly accelerated by ~34% in KO group compared to WT group at long SL (Figure 5A; Table 2).

Two-Way ANOVA revealed a significant interaction effect (*P* < 0.005) on *k*_df_ suggesting that cMyBP-C influenced the effect of SL on the rate of XB recruitment into the force-bearing state. The cause of the interaction effect was assessed using *post-hoc* tests which revealed that *k*_df_ was significantly accelerated by ~50% at short SL in WT but such an acceleration of *k*_df_ at short SL was absent in KO (Figure 5B; Table 2). Furthermore, in agreement with a previous study (Stelzer et al., 2006a), our data shows that *k*_df_ was significantly accelerated by ~38% in KO compared to WT at long SL. Thus, our stretch activation data shows that both *k*_rel_ and *k*_df_ were accelerated at short SL in WT but such trends were absent in the KO (Figures 5A,B). These findings suggest that the absence of acceleration of *k*_tr_ at short SL in the KO group (Figure 4) is due to a combined effect of the absence in the accelerations of *k*_rel_ and *k*_df_ at short SL in KO group (Figure 5). Collectively, our results suggest that cMyBP-C modulates length-dependent changes in the kinetics of XB detachment and attachment in cardiac muscle.

### Effect of cMyBP-C on length-dependent changes in the magnitude of stretch-induced increase in muscle fiber stiffness (P1)

**Figure 6:**
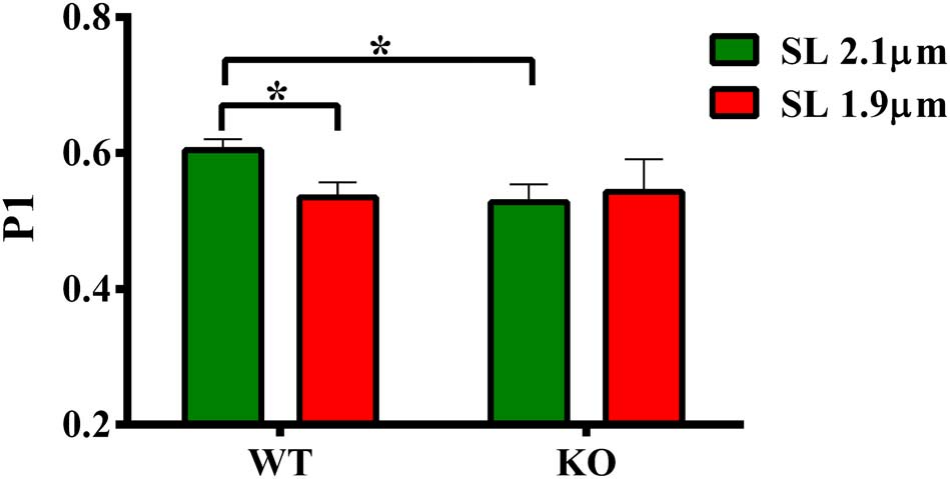
**Effect of cMyBP-C on length-dependent changes in the magnitude of sudden-stretch induced increase in muscle fiber stiffness (P1)**. P1 was calculated from the force responses elicited upon a sudden 2% stretch in muscle length imposed on isometrically-contracting ventricular preparations (Stelzer et al., 2006c). Two-Way ANOVA revealed no significant interaction effect and main effects on P1. *Post-hoc* tests showed that P1 significantly decreased at short SL vs. long SL in WT group but such a trend was absent in KO group. Also, P1 significantly decreased at long SL in KO vs. WT group. Determinations were made from 6 to 13 multicellular preparations and 3 to 4 hearts per each group. Values are reported as mean ± s.e.m. ^*^P < 0.05.

Our data shows that the XB detachment rate (*k*_rel_) was accelerated at short SL compared to long SL in WT group (Figure 5A). Also, *k*_rel_ was accelerated at long SL in KO compared to WT group (Figure 5A). We sought to determine whether such accelerations in *k*_rel_ could have arisen from a decrease in the muscle fiber stiffness because changes in *k*_rel_ can be correlated with changes in stiffness of XBs (Stelzer et al., 2006c). We imposed a sudden 2% stretch in muscle length in an isometrically-contracting muscle preparation and measured the magnitude of the elicited instantaneous increase in force (P1 in Figure 1). P1 is a result of a rapid distortion of the elastic regions of the strongly-bound XBs (Stelzer and Moss, 2006; Ford et al., 2010; Cheng et al., 2013) and is an index of the muscle fiber stiffness because both P1 and muscle fiber stiffness are well correlated to the number of parallel and force-producing XBs that are bound to actin prior to the imposed stretch in muscle length (Campbell et al., 2004; Ford et al., 2010; Cheng et al., 2013). Two-Way ANOVA revealed no significant interaction effect and main effects on P1. *Post-hoc* tests showed that P1 significantly decreased (*P* = 0.036) at short SL compared to long SL in WT group (Figure 6; Table 2). However, such a decrease in P1 at short SL was absent in KO group. Furthermore, P1 was significantly decreased (*P* = 0.032) at long SL in KO compared to WT (Figure 6; Table 2). These results suggest that a decrease in the muscle fiber stiffness contributed, at least in part, to the acceleration of *k*_rel_ observed at short SL compared to long SL in WT, and also at long SL in KO compared to long SL in WT (Figure 5A). Decreased XB stiffness could enhance strain-induced rates of XB detachment by increasing XB compliance such that XB's detach rapidly (Stelzer et al., 2006c; Cheng et al., 2013)—indicating that changes in P1 can be correlated with changes in *k*_rel_. Thus, it is likely that the absence of differences in P1 at long and short SL's in KO group (Figure 6) may have contributed to the lack of differences we observed in *k*_rel_ at long and short SL's in KO group (Figure 5A). Collectively, our data suggests that cMyBP-C modulates length-dependent changes in the rate of XB detachment via its impact on the muscle fiber stiffness.

## Discussion

Given the lack of our understanding regarding cMyBP-C's role in cardiac LDA, we performed a detailed investigation of different aspects of cardiac contractile function both in the presence and absence of cMyBP-C and at SL's 1.9 and 2.1 μm. Results from our studies demonstrate that length-dependent changes in contractile dynamics are significantly impacted in the absence of cMyBP-C in the cardiac sarcomere. Novel findings from our experiments show an attenuated length-dependent response with respect to steady-state myofilament Ca^2+^ sensitivity of force generation, and profoundly blunted length-dependent XB cycling dynamics in ventricular preparations isolated from hearts lacking cMyBP-C—suggesting that cMyBP-C is a key modulator of cardiac LDA.

### Ablation of cMyBP-C attenuates the length-dependent changes in myofilament Ca^2+^ sensitivity

An increase in myofilament Ca^2+^ sensitivity (pCa_50_) upon an increase in SL is a hallmark of LDA (Kentish et al., 1986; Dobesh et al., 2002). The effect of cMyBP-C on LDA is important to study because it is known that LDA is depressed in human hearts expressing cMyBP-C mutations (Van Dijk et al., 2012; Sequeira et al., 2013). Our results show that although the absence of cMyBP-C did not affect maximal force production (Table 1), it did attenuate the SL-mediated increase in Ca^2+^ sensitivity (ΔpCa_50_) (Figures 3A–C), a result that agrees with an earlier report (Cazorla et al., 2006). Cazorla et al reported an attenuated ΔpCa_50_ in the KO when compared to the WT myocardium (ΔpCa_50_ of 0.16 pCa units in KO vs. a ΔpCa_50_ of 0.23 pCa units in WT). In this study we found a similar trend, although here the ΔpCa_50_ was 0.11pCa units in WT skinned myocardium whereas it was 0.07 pCa units in KO skinned myocardium (Figure 3). In our study, the attenuation of ΔpCa_50_ was due to higher submaximal force production, as suggested by a higher pCa_50_, at short SL in KO vs. WT group (Figure 3C). These results indicate that the SL-based mechanisms governing myofilament Ca^2+^ sensitivity are altered in the KO group, and more so at short SL.

In view of the observation that the absence of cMyBP-C shifts the juxtaposition of the myosin heads toward the thin filament (Colson et al., 2007) thereby enhancing the probability of XB interaction, it is likely that the increased force at submaximal [Ca^2+^] at short SL in KO group could have arisen from an increased number of XBs interacting with the thin-filament. It is generally accepted that when SL is shortened, the distance between the thick- and thin-filaments increases (Rome, 1968; McDonald and Moss, 1995). It is possible that because cMyBP-C ablation inherently reduces the relative distance between actin and myosin XB's (Colson et al., 2007), the length-dependent increase in acto-myosin distance with decreased SL is diminished in the KO group, thereby, resulting in greater XB interaction and increased force production. It is also possible that the observed increase in Ca^2+^ sensitivity at short SL in the KO group may be due to an increase in the apparent Ca^2+^ binding affinity of TnC mediated by strongly-bound XBs (Pan and Solaro, 1987; Hannon et al., 1992; Moss et al., 2004). In this context, a recent *in situ* time-resolved FRET study showed that the effects of strongly-bound XBs are transmitted allosterically to the N-terminus of TnC (N-TnC) via changes in the interaction between tropomyosin (Tm) and TnI (Li et al., 2014). The net result of the feedback effect of strongly-bound XBs is to shift the Tm to the open state and also to stabilize the open conformation of the N-TnC, thereby enhancing the affinity of TnC for Ca^2+^ to cause increased force production (Li et al., 2014). Thus, enhanced XB interaction at short SL in the absence of cMyBP-C may indirectly increase the Ca^2+^ sensitivity in the KO group via a thin filament mediated mechanism.

### Ablation of cMyBP-C blunts the length-dependent changes in XB cycling kinetics

As demonstrated previously (Stelzer et al., 2006b), our present data show that *k*_tr_ was accelerated in KO compared to WT group at long SL (Figure 4). Because changes in *k*_tr_ indicate a shift in the equilibrium in the transitions between the closed to open states (McKillop and Geeves, 1993) of the thin-filament (Campbell, 1997), our data suggests that in the absence of cMyBP-C the thin-filaments are shifted more toward the open state. To understand the impact of cMyBP-C on length-dependent changes in XB transitions/cycling from weak- to strong-binding states, we measured *k*_tr_ at short and long SL's. Our data show that *k*_tr_ was accelerated at short SL compared to long SL in WT group (Figure 4) indicating the XB cycling is accelerated at short SL. In support of this observation, an earlier study showed that loaded shortening velocity and *k*_tr_ were significantly accelerated at short SL compared to long SL in skinned rat cardiac myocytes (Korte and McDonald, 2007). The mechanism for such an increase in *k*_tr_ at short SL may arise from the acceleration of XB cycling kinetics such that there are more XBs working against a constant load because of increased XB flexibility which allows XBs to radially extend toward the thin-filament at short SL (Korte and McDonald, 2007). Increased flexibility of XBs at short SL may also arise due to decreased stiffness of titin (Granzier and Irving, 1995), a consequence of which is a decreased force exerted by titin on cMyBP-C which can in turn lead to decreased constraint imposed by cMyBP-C on the myosin XBs (Korte and McDonald, 2007). Such increases in *k*_tr_ at short SL were also reported by Adhikari et al who attributed the increases in *k*_tr_ to an increased XB detachment rate at short SL (Adhikari et al., 2004).

Because *k*_tr_ encompasses both the rates of XB attachment (*f*) and detachment (*g*) (Brenner and Eisenberg, 1986), we determined if increased *k*_tr_ observed at short SL in WT was due to increase in either *f* or *g*, or both. Using length perturbation experiments (Gresham et al., 2014), we measured the rates of force development (*k*_df_) and force decay (*k*_rel_), parameters that are analogous to *f* and *g*. Our data shows that acceleration of *k*_tr_ at short SL in WT group was indeed due to a combination of increases in both *k*_df_ and *k*_rel_ (Figure 5). Our data also shows that both *k*_df_ and *k*_rel_ did not increase at short SL in KO skinned myocardium such that the values were not significantly different from those at long SL (Figure 5). A recent study (Tanner et al., 2014) showed that XB detachment rates were accelerated in papillary muscle isolated from KO hearts compared to WT hearts but only under a β-MHC background at very long SL (2.2–3.3 μm). Under an α-MHC background, XB detachment rates displayed a slight non-statistically significant increase in KO papillary muscles compared to WT papillary muscles, in contrast to the larger accelerations in XB detachment we observed in KO multicellular preparations at shorter SL (i.e., 2.1 μm), isolated from hearts expressing predominantly α-MHC. Taken together, our data shows that an absence of acceleration in *k*_tr_ at short SL in KO group was due to the absence of accelerations in both *k*_df_ and *k*_rel_ at short SL (Figure 5). Thus, our study suggests that the mechanisms influencing length-dependent changes in XB transitions between weak- to strong-binding states are blunted in the absence of cMyBP-C.

### Ablation of cMyBP-C blunts length-dependent changes in muscle fiber stiffness and cooperative mechanisms

To test whether changes in XB detachment (as assessed by *k*_rel_, Figure 5) due to cMyBP-C ablation or changes in SL were related to altered XB compliance and muscle fiber stiffness, we estimated the magnitude of the instantaneous increase in force P1, a parameter that represents the stretch-induced strain of the strongly-bound XBs and an indicator of XB stiffness (Ford et al., 2010; Cheng et al., 2013). Our measurements showed that P1 values were decreased in WT at short SL compared to long SL, and also in KO at long SL when compared to WT at long SL (Figure 6; Table 2). Because P1 can be correlated to *k*_rel_ (Stelzer et al., 2006c; Cheng et al., 2013), our results are consistent with the idea that decreased muscle fiber stiffness contributed to the observed acceleration in the XB detachment. Significantly, our results demonstrate that muscle fiber stiffness decreased at short SL compared to long SL in WT but not in KO group (Figure 6), suggesting that the lack of length-dependent changes in *k*_rel_ seen in KO group (Figure 5) may have been related to the lack of length-dependent changes in the muscle fiber stiffness (Figure 6), because muscle fiber stiffness in KO group is already significantly lower than WT group at long SL.

To test whether changes in XB recruitment, (as assessed by *k*_df_, Figure 5) due to cMyBP-C ablation or changes in SL were related to changes in cooperative mechanisms, we estimated the Hill coefficient, *n*_H_ from the pCa-tension relationships. Our estimates showed that *n*_H_ values were increased in WT at short SL compared to long SL (Figure 3D), a result that is consistent with previous studies (Ford et al., 2012; Gollapudi et al., 2012). This suggests that enhanced cooperative mechanisms may have accelerated the XB recruitment rate at short SL in WT group. This increase in *n*_H_ may be a result of enhanced Ca^2+^ binding to Tn, near-neighbor interactions among Tn-Tn, XB-Tn, and XB-XB (Razumova et al., 2000; Campbell et al., 2001). Notably, such an increase in *n*_H_ at short SL was absent in KO (Figure 3D) which may have likely contributed to the absence of length-dependent changes in *k*_df_ in the KO group (Figure 5B). In the context of the KO model, it is likely that depressed cooperative XB-XB (Razumova et al., 2000; Moss et al., 2004), XB-Tn (Razumova et al., 2000; Chandra et al., 2007) and XB-Ca^2+^/TnC (Li et al., 2014) interactions at short SL may have contributed to the blunting of the increase in *n*_H_ with decrease in SL. Therefore, our data shows that length-dependent changes in cooperative mechanisms are depressed when cMyBP-C is absent in the sarcomere.

## Conclusions

Our study provides evidence to show that cMyBP-C plays a key role in fine-tuning length-dependent cardiac contractile function via its impact on myofilament responsiveness to Ca^2+^, XB cycling kinetics, and muscle fiber stiffness. Taken together, our findings suggest that impaired LDA may contribute to depressed myocardial contractile function in human patients harboring mutations in cMyBP-C that ultimately cause a significant decrease in the amount of cMyBP-C expression in the sarcomere.

## Author contributions

Ranganath Mamidi and Julian E. Stelzer contributed to the conception and design of the experiments. Ranganath Mamidi, Kenneth S. Gresham, and Julian E. Stelzer participated in performing the experiments, data acquisition, data analysis, data interpretation, drafting, and revising the manuscript. All authors approved the final version of the manuscript.

### Conflict of interest statement

The authors declare that the research was conducted in the absence of any commercial or financial relationships that could be construed as a potential conflict of interest.
